# Ethical, Legal, and Sociocultural Issues in the Use of Mobile Technologies and Call Detail Records Data for Public Health in the East African Region: Scoping Review

**DOI:** 10.2196/35062

**Published:** 2022-06-02

**Authors:** Juliet Nabbuye Sekandi, Kenya Murray, Corinne Berryman, Paula Davis-Olwell, Caroline Hurst, Robert Kakaire, Noah Kiwanuka, Christopher C Whalen, Erisa Sabakaki Mwaka

**Affiliations:** 1 Global Health Institute College of Public Health University of Georgia Athens, GA United States; 2 Department of Epidemiology and Biostatistics College of Public Health University of Georgia Athens, GA United States; 3 Department of Health Promotion and Behavior College of Public Health University of Georgia Athens, GA United States; 4 Department of Epidemiology and Biostatistics School of Public Health Makerere University Kampala Uganda; 5 Department of Anatomy, School of Biomedical Sciences College of Health Sciences Makerere University Kampala Uganda

**Keywords:** mobile health, public health, ethics, privacy, call detail records, East Africa, Africa, mobile apps, mHealth

## Abstract

**Background:**

The exponential scale and pace of real-time data generated from mobile phones present opportunities for new insights and challenges across multiple sectors, including health care delivery and public health research. However, little attention has been given to the new ethical, social, and legal concerns related to using these mobile technologies and the data they generate in Africa.

**Objective:**

The objective of this scoping review was to explore the ethical and related concerns that arise from the use of data from call detail records and mobile technology interventions for public health in the context of East Africa.

**Methods:**

We searched the PubMed database for published studies describing ethical challenges while using mobile technologies and related data in public health research between 2000 and 2020. A predefined search strategy was used as inclusion criteria with search terms such as “East Africa,” “mHealth,” “mobile phone data,” “public health,” “ethics,” or “privacy.” We screened studies using prespecified eligibility criteria through a two-stage process by two independent reviewers. Studies were included if they were (1) related to mobile technology use and health, (2) published in English from 2000 to 2020, (3) available in full text, and (4) conducted in the East African region. We excluded articles that (1) were conference proceedings, (2) studies presenting an abstract only, (3) systematic and literature reviews, (4) research protocols, and (5) reports of mobile technology in animal subjects. We followed the five stages of a published framework for scoping reviews recommended by Arksey and O’Malley. Data extracted included title, publication year, target population, geographic region, setting, and relevance to mobile health (mHealth) and ethics. Additionally, we used the PRISMA (Preferred Reporting Items for Systematic Reviews and Meta-Analyses) Extension for Scoping Reviews checklist to guide the presentation of this scoping review. The rationale for focusing on the five countries in East Africa was their geographic proximity, which lends itself to similarities in technology infrastructure development.

**Results:**

Of the 94 studies identified from PubMed, 33 met the review inclusion criteria for the final scoping review. The 33 articles retained in the final scoping review represent studies conducted in three out of five East African countries: 14 (42%) from Uganda, 13 (39%) from Kenya, and 5 (16%) from Tanzania. Three main categories of concerns related to the use of mHealth technologies and mobile phone data can be conceptualized as (1) ethical issues (adequate informed consent, privacy and confidentiality, data security and protection), (2) sociocultural issues, and (3) regulatory/legal issues.

**Conclusions:**

This scoping review identified major cross-cutting ethical, regulatory, and sociocultural concerns related to using data from mobile technologies in the East African region. A comprehensive framework that accounts for the critical concerns raised would be valuable for guiding the safe use of mobile technology data for public health research purposes.

## Introduction

The exponential scale and pace of real-time data generated from mobile phones present new opportunities and challenges across multiple sectors, including health care delivery and public health [1]. By 2025, mobile subscribers are projected to grow from 477 million to 617 million [2]. One opportunity arising from the growth trajectory in connected mobile technology devices is the evolving field of mobile health (mHealth). The World Health Organization defines mHealth as medical and public health practice supported by mobile devices such as phones, patient monitors, smartphone apps, and functionalities, including voice or SMS text messages [3]. Globally, mHealth solutions have been deployed to address problems such as a shortage of health care workers [4,5], monitoring medication adherence [6,7], and improving quality of health care [8].

In 2020, the National Institutes of Health (NIH) announced a grand opportunity for health research for new funding, titled “Harnessing Data Science for Health Discovery and Innovation in Africa (DS-I Africa).” The goal is to spur new health discoveries and catalyze innovation in health care, public health, and health research in Africa through data science [9]. Additionally, the DS-I Africa announcement called for research to explore the ethical, legal, and social implications associated with data science [10]. These calls highlight new areas for scientific inquiry and recognize the need to address new ethical challenges when using “big data” streams across Africa.

The use of mobile phone call detail records (CDRs), which are time-stamped activity logs that are also known as billable events, is a relatively new concept in health research [11-14]. Although telecommunication companies have used CDRs for more than three decades for customer billing and targeted marketing purposes, the business sector has only recently innovated more ways to use these data in remote banking and financial service delivery. However, the health sector has sparsely leveraged such enormous amounts of mobile phone data for public health, especially in Africa [3-5]. The Data for Development Challenge, Google Flu Trends, Datathon for Social Good, and the Cairo Transport App Challenge, among others, have used archived CDRs to study human activity [6]. In the field of infectious diseases, innovative use of aggregated anonymized data from CDRs revealed informative patterns of the spatiotemporal transmission of various pathogens in the community, including rubella [15,16], malaria [12,17,18], and Ebola [19,20] in Africa. More recently, the COVID-19 pandemic has accelerated this novel utilization of mobile technologies and CDRs for public health surveillance and research [21-23]. However, a lack of well-established standardized ethical and regulatory frameworks has limited the widespread use of such data in health research, particularly in East Africa [24].

Our ongoing NIH-funded Mapping Tuberculosis Transmission Study (MATTS) in Kampala, Uganda, motivated this scoping review. MATTS takes a unique approach that utilizes individual-level CDRs combined with epidemiologic surveys and molecular data to map potential tuberculosis transmission “hot spots.” During the initiation of the study, members of the research team from the University of Georgia and Makerere University met with key stakeholders representing the public and private sectors in Uganda. The private sector players were telecommunication companies, and the public players were government agencies or regulatory entities. The goal of the meetings was to learn about existing ethical standards and policy guidelines that apply to utilizing CDRs and other data from mobile technologies for health research. The meetings led to several questions about data access, sharing, transfer, storage, security, privacy, confidentiality, legal, regulatory, and social concerns. In the end, our team surmised that the government and telecommunication entities had existing policies and procedures on personal data that did not necessarily accommodate the utilization of mobile data such as CDRs for health research. Therefore, our research serves as a logical step to generate evidence that is critical for informing the development of policy frameworks that will facilitate better access to mobile data for public health research.

In utilizing CDRs, there are potential points of ethical breaches as personal information flows from a user’s mobile phone to a database for health research purposes. For example, when a user activates a connection to mobile phone networks by calling or texting, “event-driven” personal data are generated in real time and stored as CDRs, whereas “network-driven” data are generated when the cell phone is not in active use (see Multimedia Appendix 1). The event-driven metadata in the records contain the caller and recipient phone numbers, date and time of the call, call duration, and the coordinates of the geographical location of the cell tower that serviced the billable event [25]. Each of the data points generated can potentially result in privacy breaches if the data are accessed without ethical safeguards [26]. The Belmont Report provides standard bioethics guidance for researchers based on the core ethical principles (ie, respect for persons, beneficence, and justice) [27]. Even so, we expect that there are new ethical challenges related to the use of the data generated from mobile phones or other technology/devices that warrant further evaluation.

We situated this scoping review in the East African region because of the cross-cutting similarities in sociocultural context, the landscape and stage of mobile technology infrastructure development, and the likelihood of future unified regulatory policies given shared economic interests. Scoping reviews are useful for examining emerging evidence when it is still unclear what other, more specific, questions can be addressed by a more precise systematic review [28]. The objective of the scoping review was to explore the ethical and related concerns that arise from the use of data from CDRs and mobile technology interventions for public health in the context of East Africa.

Our review findings are used to inform an ongoing primary study to explore ethical, legal, sociocultural, and regulatory concerns from the perspective of key stakeholders in the public and private sectors in Uganda. We expect that this work will further inform the development of comprehensive guidelines relevant to mHealth and public health research in the East African region.

## Methods

### Design

We followed five of the six stages of the framework for scoping reviews proposed by Arksey and O’Malley [29]: (1) identifying the research question; (2) identifying relevant studies; (3) study selection; (4) charting the data; and (5) collating, summarizing, and reporting the results. The sixth stage of the framework is optional, which was not included in this scoping review. It involves a consultation exercise with stakeholders to gain a more holistic view of the issues under evaluations. Additionally, we used the PRISMA (Preferred Reporting Items for Systematic Reviews and Meta-Analyses) Extension for Scoping Reviews checklist to guide the presentation of this scoping review [30]. We applied existing definitions to identify common ethical concerns related to informed consent, privacy and confidentiality, and data security and protection. We also used team consensus to classify concerns that fit within legal, regulatory, and sociocultural categories.

### Operational Definitions

Privacy of health information refers to an individual’s right to control the acquisition, uses, or disclosures of his or her identifiable health data for others [31,32]. Confidentiality refers to the obligations of those who receive information to respect the privacy interests of those to whom the data relate [31,32]. Data security refers to the physical, technological, or administrative safeguards or tools used to protect identifiable health data from unwarranted access or disclosure [32]. Data protection is closely related to data security, which refers to adopting appropriate, reasonable, technical, and organizational measures to prevent loss, damage, or unauthorized destruction or processing of personal data [33]. General ethical concerns refer to research processes that fail to uphold or violate the principles of respect for persons, balance of potential benefits and harms, and equitable sharing of benefits and burdens across research groups [27].

### Identification of the Research Question

The primary research question was: *what are the ethical, privacy, and data security concerns pertaining to the use of mHealth technologies and mobile phone data for public health research in the East African region?* The rationale for focusing on the five countries in East Africa was that the ethics and mHealth technology landscapes are likely to be similar in several ways within the same region. Therefore, the findings could inform regional guidelines and policies.

### Identification of Relevant Studies

A systematic search of the literature was performed on November 18, 2020, in the PubMed database using the search terms “mobile,” “ethics,” and “East Africa.” The detailed search strategy and terms used are provided in Textbox 1. The search was limited to English and included only studies focused on the East Africa region. We considered only articles published from 2000 to 2020 due to the widespread use of mobile technology during this period in the region of interest.

**Full search strategy of the scoping review in the PubMed database.**

(“Digital Health” [Title/Abstract] OR “Telemedicine” [MeSH (Medical Subject Heading)] OR “Cell Phone” [MeSH] OR “Smartphone” [MeSH] OR “mHealth” [Title/Abstract] OR “telemedicine” [Title/Abstract] OR “remote monitoring” [Title/Abstract] OR “mobile technology” [Title/Abstract] OR “mobile health” [Title/Abstract] OR “video observed therapy” [Title/Abstract] OR “video observed treatment” [Title/Abstract] OR “video directly observed treatment” [Title/Abstract] OR “video directly observed therapy” [Title/Abstract] OR “Call Detail Records” [Title/Abstract] OR “cell phone” [Title/Abstract] OR “cell phone” [Title/Abstract] OR “mobile phones” [Title/Abstract] OR “smartphone” [Title/Abstract] OR “cellphone data” [Title/Abstract] OR “cell phone data” [Title/Abstract] OR “data privacy” [Title/Abstract] OR “cell phone/ethics” [MeSH] OR “Cell Phone/legislation and jurisprudence” [MeSH] OR “Cell Phone Use/legislation and jurisprudence”[Mesh] OR “Cell Phone Use/therapeutic use”[Mesh]) **AND** (“bioethics” [MeSH] OR “ethics” [Mesh] OR “bioethics” [Title/Abstract] OR “public health ethics” [Title/Abstract] OR “ethics” [Title/Abstract] OR “research ethics” [Title/Abstract] OR “autonomy” [Title/Abstract] OR “personal autonomy” [MeSH] OR “principle-based ethics” [MeSH] OR “personal autonomy” [Title/Abstract] OR “principle-based ethics” [Title/Abstract] OR “privacy” [MeSH] OR “confidentiality” [MeSH] OR “computer security” [MeSH] OR “privacy” [Title/Abstract] OR “confidentiality” [Title/Abstract] OR “computer security” [Title/Abstract] OR “data anonymization” [Title/Abstract] OR “data compromising” [Title/Abstract] OR “compromising of data” [Title/Abstract] OR “data security” [Title/Abstract] OR “information protection” [Title/Abstract] OR “Data Protection” [Title/Abstract] OR “Data Security” [Title/Abstract] OR “information protection” [Title/Abstract] OR “Data encryption” [Title/Abstract] OR “Data encryptions” [Title/Abstract] OR “social justice” [Title/Abstract]) **AND** (“Africa” [MeSH] OR “Africa, South of the Sahara” [MeSH] OR “Africa, Eastern” [MeSH] OR “Tanzania” [MeSH] OR “Rwanda” [MeSH] OR “Burundi” [MeSH] OR “Kenya” [MeSH] OR “Uganda” [MeSH] OR “Tanzania” [ALL] OR “Rwanda” [ALL] OR “Burundi” [ALL] OR “Kenya” [ALL] OR “Uganda” [ALL])

### Study Selection

Retrieved studies reporting on mobile technology and health in the East African region were included for further review. Two independent reviewers for each article applied the inclusion and exclusion criteria, with an additional author serving as the tiebreaker in the event of uncertainty regarding whether an article met the inclusion or exclusion criteria. Studies were included if they were (1) related to mobile technology and health, (2) published in English from 2000 to 2020, (3) available in full text, and (4) conducted in the East African region. We excluded articles that (1) were conference proceedings, (2) without full text or presenting an abstract only, (3) systematic and literature reviews, (4) research protocols, and (5) reports of mobile technology in animal subjects.

### Charting of the Data

Two authors (CH and CB) performed abstract screening. Three authors (KM, CH, and CB) subsequently reviewed full-text articles, and extracted and stored the data in a Microsoft Excel database developed for this review. From the eligible studies, we extracted the title, publication year, journal name, impact factor, target population, geographic region, setting, and relevance to mHealth and ethics. For each stage of the review, two reviewers (KM and CB) independently reviewed each full-text article. Discrepancies were discussed collectively until consensus was reached.

### Collating, Summarizing, and Reporting Results

Given the diversity of articles included in the final scoping review, we employed an iterative team discussion approach to the analysis in order to establish the main themes related to ethical and related concerns. We use descriptive statistics to summarize the characteristics of the studies, including country of publication, year of publication, area of focus, study design, and mHealth intervention employed.

## Results

### Articles Selected

**Figure 1:**
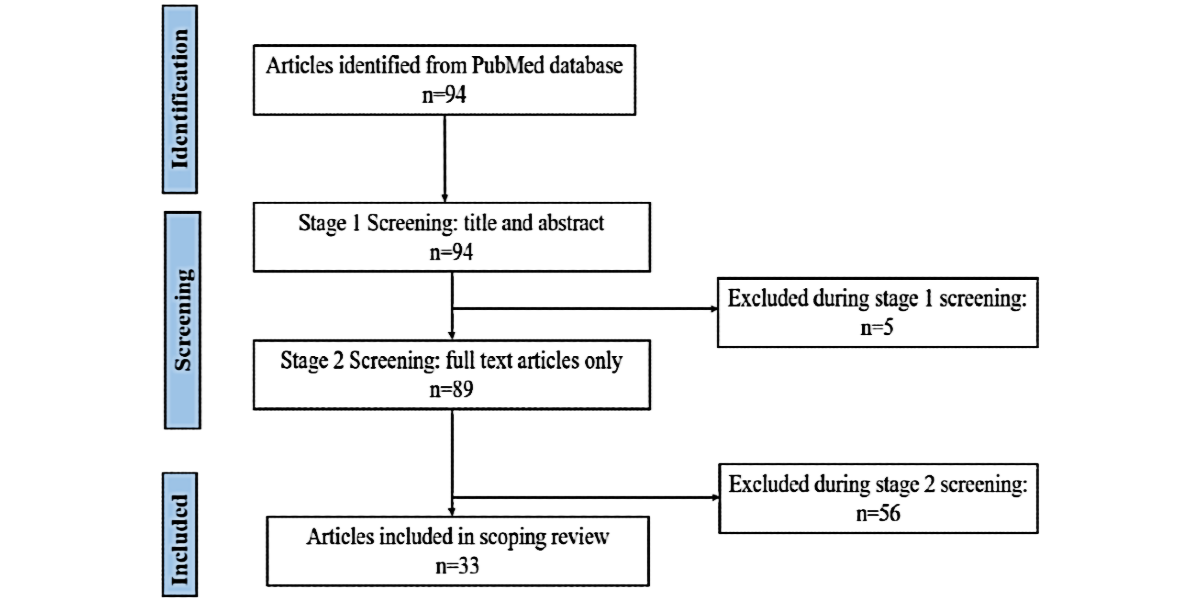
PRISMA (Preferred Reporting Items for Systematic Reviews and Meta-Analyses) flow diagram showing article search and screening.

The search identified 94 articles eligible for further review (Figure 1). Of these, 59 did not meet the inclusion criteria and 5 additional studies were excluded based on a review of titles and abstracts. We included 33 studies published in 22 journals in the final scoping review.

### Study Characteristics

The 33 articles retained in the final scoping review represent studies conducted in three out of five East African countries, including 14 (42%) from Uganda and 13 (39%) from Kenya (Table 1). The number of studies published on ethical concerns of mHealth technology use and mobile phone data increased over time, beginning with one in 2009 and climbing to six in 2018. The articles were mostly published in the journal *AIDS and Behavior*, followed by *Journal of Medical Internet Research* and *BMC Public Health*. The majority of mHealth interventions were SMS-based, followed by mobile phone app–based, whereas CDR aggregate data analysis was seldom used among the studies we reviewed. More than half of the studies included were observational in study design, followed by a mixed methods design. Two of the 33 studies included met the eligibility criteria but did not mention ethical concerns beyond compliance with human research and approval from respective institutional review boards.

Three main categories of concerns related to the use of mHealth technologies and mobile phone data can be conceptualized as (1) *ethical issues* (adequate informed consent, privacy and confidentiality, data security and protection), (2) *sociocultural issues*, and (3) *regulatory/legal issues*. These themes are not necessarily mutually exclusive. A detailed summary of each study by theme is provided in Table 2. Ethical concerns were predominantly identified in the included studies, which are discussed in detail below.

**Table 1:** Summary of articles included in the scoping review for final analysis (N=33).

Characteristics		Studies, n (%)
**Country**		
	Uganda	14 (42)
	Kenya	13 (39)
	Tanzania	5 (15)
	Uganda and Tanzania	1 (3)
**Publication year**		
	2009	1 (3)
	2010	1 (3)
	2011	1 (3)
	2012	2 (6)
	2013	4 (12)
	2014	1 (3)
	2015	3 (9)
	2016	4 (12)
	2017	5 (15)
	2018	6 (18)
	2019	5 (15)
**mHealth^a^ intervention**		
	SMS messaging	14 (42)
	Mobile phone app	5 (15)
	Mobile phone	4 (12)
	CDR^b^ aggregate analysis	2 (6)
	Voice and SMS messaging	2 (6)
	Mobile phone survey	2 (6)
	Tablet	2 (6)
	Computer-assisted personal interviewing	1 (3)
	Telemedicine	1 (3)
**Study design**		
	Observational	23 (70)
	Mixed methods	4 (12)
	Descriptive report	2 (6)
	Modelling	2 (6)
	Quasiexperimental	1 (3)
	Randomized controlled trial	1 (3)
**Area of focus**		
	HIV/AIDS	15 (45)
	Reproductive health	4 (12)
	Infectious disease	3 (9)
	Noncommunicable disease	3 (9)
	Eye and vision health	2 (6)
	Maternal and child health	2 (6)
	Data management	1 (3)
	Image-based health	1 (3)
	Telemedicine	2 (3)

^a^mHealth: mobile health.

^b^CDR: call detail record.

**Table 2:** Descriptive themes identified regarding ethical, legal, regulatory, and sociocultural concerns of mobile health (mHealth) interventions and mobile phone data use.

Studies and countries		Domain of concern	mHealth intervention	Key recommendation
**Adequate informed consent**				
	Uganda [34,35], Kenya [36]	Cannot consent due to failed understanding of technology	Computer-assisted personal interviewing, mobile phone surveys, telemedicine	Participants’ inadequate understanding of the capabilities of mHealth interventions; thus, the question of whether they understood sufficiently to properly consent was raised
	Tanzania and Uganda [37]	Consent must be a prerequisite to mHealth interventions	Image-based mHealth app	Not provided
	Kenya [38]	Consent needed for different types of prevention of mother-to-child transmission information	SMS text messaging	Not provided
**Privacy and confidentiality**				
	Uganda [39-48]	Password/PIN^a^ protection	SMS text messaging, mobile job aid, mobile phone tool	Use of PIN and passwords offers protection of confidentiality. However, the mere presence of passwords may arouse suspicion by intimate partners and others
	Kenya [38,49-53]	Phone theft, data breaches	Smartphone app	Not provided
	Tanzania [16,18,54,55]	Phone sharing	Smartphone app	Not provided
**Data security and protection**				
	Tanzania [56-58]	Data breaches, phone theft, access rights to protect client data	Smartphone app, mobile job aid, mobile app	mHealth interventions should have an eye toward maternal perception of data security, and with prior and ongoing consultation with community members. Locking phones with a password improves the ability of CHWs^b^ to maintain confidentiality of their clients’ information, particularly for women who did not want to disclose their use of family planning to their husband or other family members
	Kenya [50,59]	Phone theft	SMS text messaging, smartphone ophthalmic exam	Not provided
	Uganda [34,35]	Mobile phone numbers linked to national ID cards	Computer-assisted personal interviewing, interactive voice survey	Not provided
**Sociocultural**				
	Tanzania [56,60]	Breach of pregnancy-related information	Mobile phone app	Support from male heads of household may be important in implementing successful mHealth interventions
	Uganda [43]	Gender dynamics, delivery of interactive voice survey in voice of opposite sex	Interactive voice survey, SMS messaging	Preference of male or female voices for phone call interventions may vary based on the patient’s gender
**Regulatory/legal**				
	Tanzania [56]	Data protection legislation	Mobile phone app	Data protection legislation is needed in regions where local dynamics are important when protecting individuals’ health data
	Uganda [35]	National ethics guidelines	Interactive voice survey	Not provided

^a^PIN: personal identification number.

^b^CHW: community health worker.

### Ethical Concerns

#### Adequate Informed Consent

Five studies (15%) described adequate consent concerns pertaining to mHealth technology use. In Uganda, participants feared that computer-assisted personal interviews to collect maternal health data also involved audio recording of their responses. To address this concern, the researchers reiterated the need to ensure that participants clearly understand how the technology works and answer any questions during the consent process [34]. In addition, Mwaka and colleagues [35] echoed the importance of understanding consent requirements and how local cultural norms impact participation in mobile phone–based surveys.

In Kenya, researchers questioned the validity of informed consent when there are English words that have no direct equivalent translation in a native local dialect such as Kiswahili. For example, one study [36] found that more than half of the participants did not understand several translated words such as “videoconference,” “store and forward,” “digital photograph,” “wireless,” or “email” in either language. Thus, a combination of words was needed to explain telemedicine terms; nevertheless, the participants seemed to have a poor understanding, thereby threatening the validity of the consent process [36]. Jennings and colleagues [38] further pointed out the need to include a standardized technology orientation for prevention of mother-to-child transmission (PMTCT) to ensure adequate informed consent. The technology orientation should be applicable to all users regardless of their specific health condition.

#### Privacy and Confidentiality

Among the 33 included studies, 22 (67%) described privacy and confidentiality concerns regarding utilizing mHealth technology and mobile phone data. Specifically, two studies were not focused directly on end-user experiences regarding privacy and confidentiality, but highlighted the need for additional research on how mHealth technologies could improve patient privacy [61,62].

Four studies conducted in Uganda explored an SMS-based intervention to support care among people living with HIV/AIDS (PLWHA) and revealed mixed results. Some participants had no privacy concerns related to disclosure of their personal health–related information, including their HIV status [39-42]. For example, participants believed that using personal identification numbers or passwords increased protection of their individual data. End users completing health surveys using interactive voice response (IVR) technology in Uganda also indicated that their confidentiality was increased by using mobile phone–based surveys [43].

Moreover, there were increased privacy concerns in situations where the participants had not disclosed their HIV status to family members or friends, especially when a phone was shared. Three studies in Uganda described privacy and confidentiality concerns pertaining to the use of SMS-based reminders for medication adherence, fearing that they could result in unintended HIV status disclosure when messages are seen by others [44,45,54]. Similar privacy and confidentiality concerns were expressed among pregnant HIV-infected women in Kenya indicating opposition to receiving messages with HIV-related terms such as “infection” or “medication,” in fear that it would disclose their HIV status [49]. Additionally, perceptions of data privacy may differ between health care workers and patients. Two studies in Uganda focused on the impact of using mHealth interventions to improve care among PLWHA. The community health workers (CHWs) expressed concerns of threat to data confidentiality if the phone is stolen or multimedia capabilities are misused, while the participants perceived the smartphone to protect their confidentiality [46,47].

In Kenya, end users of mHealth technologies supporting PMTCT, antiretroviral therapy (ART) adherence, and family planning indicated that their personal privacy was protected while discussing sensitive health matters via call or text compared to face-to-face encounters [38,50-52]. Similarly, two other studies in Kenya [55] and Tanzania [53], respectively, found minimal confidentiality concerns among participants using a smartphone to support family planning services and HIV testing. Moreover, participants indicated that they were more likely to answer questions honestly using the mHealth intervention compared to speaking in person with a provider or attending a clinic [53,55].

Aggregated and anonymized CDR data may present minimal ethical and privacy concerns when compared to individual-level or other mobile phone data. Tatem and colleagues [18] analyzed aggregate-level CDRs in Tanzania to inform malaria elimination. The researchers did not explicitly explore privacy and confidentiality concerns, but they noted that aggregated and anonymized CDRs could support analysis without compromising the privacy of the mobile phone users. Wesolowski and colleagues [16] also used aggregated and anonymized mobile phone data in the form of CDRs combined with community health surveys in Kenya to study the impact of travel on the transmission dynamics of malaria. Again, the researchers did not examine privacy concerns directly, but pointed out privacy concerns as a potential barrier to the availability of CDR data for health research.

#### Data Security and Protection

Seven of the 33 studies (21%) described data security and protection concern among users. Two studies in Tanzania described mixed levels of data security and protection concerns between CHWs and their female clients during the use of mHealth interventions to improve the quality of maternal and family planning services [56,57]. CHWs had positive views when using smartphones to collect reproductive health data from their clients. They noted that using mobile technology improved data security and protection of clients’ privacy. However, some of the female clients expressed concerns about potential data breaches if personal information related to their pregnancy is stored on the smartphone and accidentally shared. In Kenya, a few data security and protection concerns were reported when researchers analyzed the perceptions of HIV-infected patients toward a mobile phone messaging intervention to support ART adherence [50]. In rural Uganda, Mercader and colleagues [34] assessed the acceptability of computer-assisted personal interviewing for maternal, newborn, and child health surveys, and concluded that respondents perceived personal health data recorded on tablets as more secure than paper surveys.

In Uganda, the new requirement to link the National Identification Number (NIN) with one’s personal phone number is perceived as a threat to data privacy and security. Mwaka and colleagues [35] explored the views of 14 key stakeholders regarding the potential challenges for obtaining consent during implementation of mobile phone surveys for IVR-based noncommunicable disease research. Some key informants highlighted overlapping concerns regarding data security and privacy where the identity of the respondent can be traced through the linkage between the NIN and phone number. In Kenya, health care providers and stakeholders expressed data security and protection concerns in using a smartphone-based ophthalmic exam, recommending a secure data encryption system to protect personal data [59].

In Tanzania, Steiner and colleagues [58] reviewed four field applications of a mobile device deployed to support low-resourced countries with data entry and project monitoring. The researchers created a data collection platform with user-defined access rights to ensure the data security and protection of clients [58]. During a 3-day workshop focused on addressing ethical issues of safety and privacy among mHealth developers and users in low-resource settings, the authors gathered solutions from 27 mHealth stakeholders from various geographical regions, including Uganda and Tanzania [37]. The stakeholders concluded that patient authorization and informed consent must remain prerequisites to mHealth interventions, specifically during the implementation and scale-up stages.

### Sociocultural Issues

Three mHealth studies (9%) described concerns related to complex gender dynamics and sociocultural beliefs. In Tanzania, two studies revealed gender-based power imbalances among women and men. Female participants reported low mobile phone ownership and expressed concerns about spouses being suspicious that if a wife owned a phone, it may facilitate infidelity in the relationship [56,60]. In Uganda, female participants expressed sociocultural concerns pertaining to using mHealth interventions to support health. For example, some participants were reluctant to use IVR for health purposes because of community beliefs that there are “*evil spirits*” that could claim the life of the recipient if one received unknown calls. In addition, women participants were particularly apprehensive about IVR surveys being sent to their cell phones if they were being administered in a male voice; they feared that this would raise suspicion of cheating by their spouses and could spark domestic violence [43]. Phone sharing is particularly common in countries within Africa, thus creating another potential source of privacy breaches and sociocultural concerns.

### Regulatory and Legal Issues

Two studies raised regulatory and legal concerns regarding using mHealth technology. These studies reflect the perspectives of the researchers governing the studies and the key stakeholders surveyed. In Tanzania, researchers concluded that the findings of their study on reproductive health provided impetus for stronger data protection legislation in regions such as rural Tanzania [56]. Protecting individuals’ health data is particularly important given the inherent sociocultural beliefs surrounding the secrecy of health information [56]. In Uganda, researchers indicated that the processes for mobile phone surveys should follow standard international regulatory guidelines for sharing personal information while also ensuring alignment with existing local laws and policies [35].

## Discussion

### Principal Findings

The aim of this scoping review was to synthesize the current state of evidence on the ethical, sociocultural, legal, and regulatory concerns related to the use of mHealth technologies and mobile phone data for public health research in the East Africa region. Our review builds on a growing body of work examining concerns with mHealth data security, privacy, and confidentiality [24]. To our knowledge, this is the first study to synthesize end-user and public health research concerns pertaining to mobile phone data use in the East Africa region. Five interrelated themes emerged as key concerns with the use of mobile phone data and mHealth interventions: adequate informed consent, privacy and confidentiality, data security and protection, sociocultural issues, and regulatory/legal issues. Of equal concern is the collection, use, and sharing of personal information such as CDRs to third parties without the notice or consent of consumers. Countries need to put in place legislation to protect the privacy and secure these personal data while facilitating their use for the good of the populations. In the future, these themes and others could inform the formulation of a framework for ethical regulatory policies using mHealth in the East African Community (EAC) region.

African countries have rapidly adopted the use of mobile technologies to support their daily needs such as social, economic, education, and travel needs, among many others. Specifically, the East African region shows a dominance in the adoption of mHealth programs to overcome some structural barriers in the health system [63]. Studies in high-income countries deploying mHealth interventions have revealed concerns mostly related to data privacy, security, storage, and transmission [64-66]. In contrast, most studies in the East African region highlight concerns about the potential disclosure of personal health information at the user interface, such as reading text messages. The pace of development and access to mobile technology could partly explain these differences in concerns between high- and low-income settings.

Sociocultural issues seem to influence the differential levels of positive and negative perceptions around data privacy and confidentiality concerns by gender and user group across the East African region (see Multimedia Appendix 2). For example, privacy and confidentiality concerns in Uganda and Tanzania highlighted gender norms and cultural beliefs that exist around secrecy about pregnancy and childbirth due to a fear of supernatural powers that could cause harm to the mother and unborn child. These beliefs could inhibit the willingness to share pregnancy-related personal information via the phone [67,68]. In addition, the issues of phone sharing due to limited mobile phone ownership among women could precipitate sociocultural tensions. For example, spousal conflicts and violence, especially directed toward female mHealth users, have been reported when women receive various forms of health support from male health care workers. Although mHealth interventions are beneficial, implementers must be mindful to anticipate the issues that may result from phone sharing in the cultural context of Africa [69].

mHealth data have the potential to enhance or jeopardize privacy. For example, the HIV-related stigma and discrimination among PLWHA in Africa could be attenuated if disclosure of HIV status is kept confidential. The findings of this scoping review suggest that among PLWHA mHealth interventions, supporting patient care should be accompanied by unique personal identification numbers or passwords to facilitate protection of privacy and confidentiality of HIV-related information. Several studies from Kenya and Uganda revealed that populations experienced difficulty in understanding the capability of mHealth interventions, which in turn raises questions about the informed consent process [34-36].

The lack of comprehensive ethical frameworks to guide the use of mHealth in public health research and practice remains a challenge across countries in the EAC region [70]. Very few studies included in this review explored the legal concerns of data use beyond pointing to the need for legal and regulatory policies for data protection and privacy of personal data. In 2018, MEASURE Evaluations published a report of guidelines developed for Kenya and Tanzania to help mHealth program managers and Ministry of Health officials systematically address mHealth data privacy and security issues [24]. The authors acknowledged that the guidelines were limited in scope, only serving as a building block that would allow stakeholders to be informed, and guide the teams responsible for developing and implementing responsible data practices, especially data security and privacy.

Legal regulation of personal health information within the EAC is not uniformly developed. The Republic of Uganda [33] and the Republic of Kenya [71] passed their data protection laws in 2019, whereas Rwanda passed a similar law in 2021 [72]. Tanzania currently has a draft data protection bill that has not yet passed into law, whereas Burundi does not have a law that specifically regulates personal data protection. Based on the experience of our health research team in Uganda, the new law is not yet operationalized into clear policy guidance, particularly in the context of public health research and practice. As such, we have encountered barriers to access CDR data from telecommunication companies even when we present written and signed consent from the cell phone owners. To the greatest extent possible, each nation needs to cooperate to set up regional mobile technology standards reflecting its local situations and government policy regulations [65]. The results of our scoping review serve to highlight concerns and gaps that must be addressed to create an enabling policy and regulatory environment for public health researchers. Additionally, public views on the use of CDRs in health research must also be explored.

### Future Implications for Public-Private Partnerships

Our work has implications for public-private partnerships, given the potential mutual benefits from CDRs, a largely untapped emerging data type that is collected by telecommunication companies in the EAC region. First, the public and private sectors could share expert human capital financial and infrastructural resources to catalyze the growth of these entities within the region. For example, the public health research enterprise could quickly access a large quantity of rich data on spatial and temporal mobility patterns of the population, which are routinely collected but underutilized by telecommunication companies. This approach to collecting specific data would likely be far more efficient and cost-effective than traditional methods. Second, the private sector could gain new business insights about the populations they serve based on a range of geospatial or data-mining analyses and interpretations generated by public researchers and data scientists. In Africa, many lessons can be learned from major innovations in banking and agriculture that have already spawned from the extensive digital infrastructure. Lastly, we envision a unique opportunity for the public health and private sectors to engage jointly with regulatory policymakers to advance data governance policies for mutual benefit. It is important to note that this process will likely be dynamic given the rapidly evolving mobile technology landscape.

### Limitations

This scoping review has some limitations. First, we acknowledge that we might have missed studies relevant to mHealth and ethical, legal, or privacy concerns in the East Africa region if they were published in electronic databases other than PubMed, in a language other than English, or outside of the period of our study. Second, studies published from Rwanda and Burundi may have been excluded from this review specifically because English was specified as the only language for articles that met other inclusion criteria. In this case, any published articles written in French could have been excluded; however, during our search, we did not identify any such articles in the initial screening. Overall, we believe that the majority of the public health research articles are accessible through PubMed. Therefore, we do not expect that our findings and conclusions were significantly influenced by any papers we might have missed.

### Conclusions

This scoping review identified major cross-cutting ethical, regulatory, and sociocultural concerns related to use of data from mobile technologies in the East African region. A comprehensive framework that accounts for ethical, sociocultural, legal, and regulatory concerns in the cultural context of the EAC region is needed to guide the safe use of mobile technology data for public health research purposes.

## Appendix

Multimedia Appendix 1 Data sharing pathways for mobile phone records in mHealth and public health research.

Multimedia Appendix 2 Positive and negative perceptions about privacy, confidentiality, and safety of data collected with mobile phones.

## Abbreviations

ART: antiretroviral therapy
CDR: call detail record
CHW: community health worker
DS-I Africa: Harnessing Data Science for Health Discovery and Innovation in Africa
EAC: East African Community
IVR: interactive voice response
MATTS: Mapping Tuberculosis Transmission Study
mHealth: mobile health
NIH: National Institutes of Health
NIN: National Identification Number
PLWHA: people living with HIV/AIDS
PMTCT: prevention of mother-to-child transmission
PRISMA: Preferred Reporting Items for Systematic Reviews and Meta-analyses
