# Supplementary material for: Ethical, Legal, and Sociocultural Issues in the Use of Mobile Technologies and Call Detail Records Data for Public Health in the East African Region: Scoping Review
Source: Interact J Med Res. 2022 Jun 2;11(1):e35062. doi: 10.2196/35062 (PMC9204580; doi:10.2196/35062)

Appendix 1. Data sharing pathways for mobile phone records in mHealth and public health research


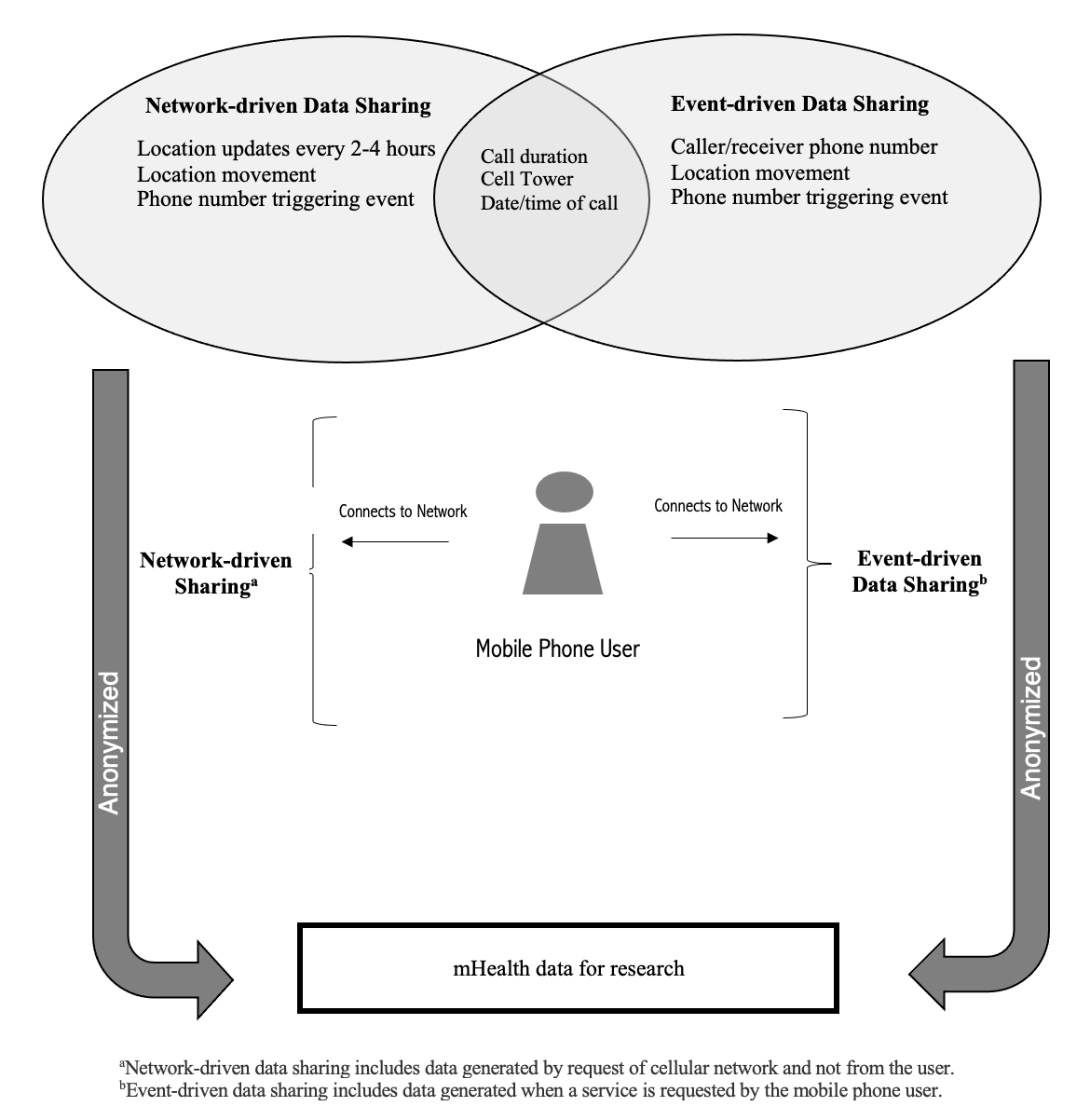

Supplement: Multimedia Appendix 1 [file ijmr_v11i1e35062_app1.docx]
