# Supplementary material for: Ethical, Legal, and Sociocultural Issues in the Use of Mobile Technologies and Call Detail Records Data for Public Health in the East African Region: Scoping Review
Source: Interact J Med Res. 2022 Jun 2;11(1):e35062. doi: 10.2196/35062 (PMC9204580; doi:10.2196/35062)

Appendix 2. Positive and negative perceptions about privacy, confidentiality and safety of data collected with mobile phones


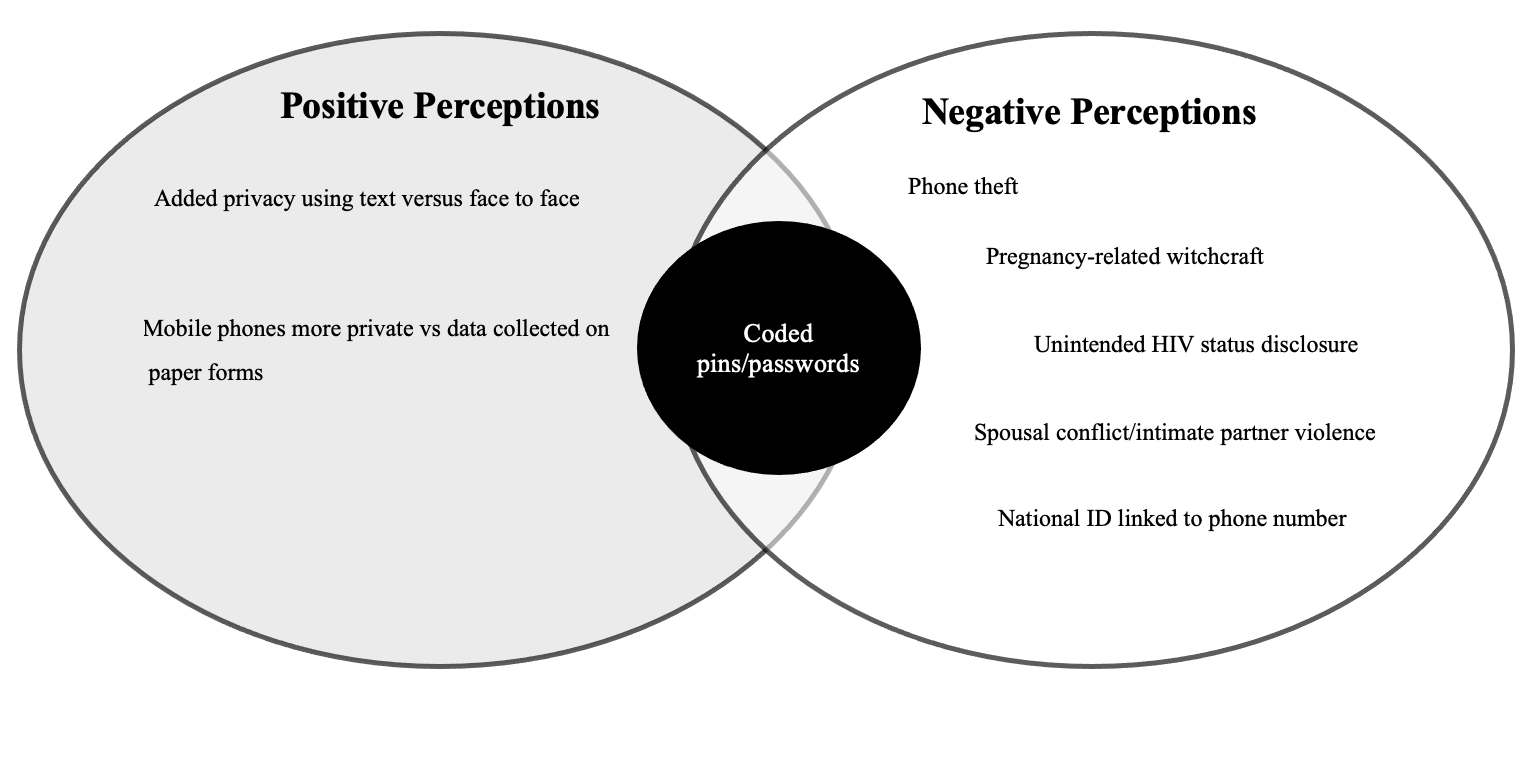

Supplement: Multimedia Appendix 2 [file ijmr_v11i1e35062_app2.docx]
